# Impact of homogeneous and heterogeneous reactions in the presence of hybrid nanofluid flow on various geometries

**DOI:** 10.3389/fchem.2022.1032805

**Published:** 2022-10-18

**Authors:** Izharul Haq, R. Naveen Kumar, Rana Gill, J. K. Madhukesh, Umair Khan, Zehba Raizah, Sayed M. Eldin, Nattakan Boonsatit, Anuwat Jirawattanapanit

**Affiliations:** ^1^ College of Sciences and Human Studies (CSHS), Prince Mohammad Bin Fahd University, Al Khobar, Saudi Arabia; ^2^ Department of Studies and Research in Mathematics, Davangere University, Davangere, India; ^3^ Department of Mechatronics, University Centre for Research and Development, Chandigarh University, Mohali, India; ^4^ Department of Mathematical Sciences, Faculty of Science and Technology, Universiti Kebangsaan Malaysia, UKM, Selangor, Malaysia; ^5^ Department of Mathematics and Social Sciences, Sukkur IBA University, Sukkur, Pakistan; ^6^ Department of Mathematics, College of Science, King Khalid University, Abha, Saudi Arabia; ^7^ Center of Research, Faculty of Engineering, Future University in Egypt, New Cairo, Egypt; ^8^ Department of Mathematics, Faculty of Science and Technology, Rajamangala University of Technology Suvarnabhumi, Nonthaburi, Thailand; ^9^ Department of Mathematics, Faculty of Science, Phuket Rajabhat University (PKRU), Phuket, Thailand

**Keywords:** hybrid nanofluid, porous medium, homogeneous and heterogeneous reactions, heat source/sink, cone, wedge and plate

## Abstract

The current work investigates the influence of porous media, homogeneous and heterogeneous reactions, and a heat source/sink on the hybrid nanoliquid circulation on three distinct surfaces (cone, plate, and wedge). The system of equations that describe the circulation issue and operating conditions is reduced to ordinary differential equations (ODEs) by using the proper similarity transformations. The Runge–Kutta–Fehlberg 45 order and the shooting approach are used to generate the numerical results. Graphs are used to show how various dimensionless limits affect the associated profiles. The results demonstrate that, in the presence of heat source/sink and porous medium characteristics, respectively, fluid velocity and heat dispersion are high in plate geometry and lower in cone geometry. The concentration profile shows the declination in the presence of both homogeneous and heterogeneous reaction intensities. The surface drag force decreases and the rate of heat dispersion rises with the addition of a porous attribute. Furthermore, cones sprinkle the heat more quickly than wedges, which disperse heat more slowly.

## Introduction

A nanofluid (NF) is a mixture of nanosized particles and a base liquid. A colloidal dispersion of nanosized particles in a base liquid is employed to make nanofluids. Thermal conductivity is poor in these basic fluids. Nanosized particles are used for the long-term effectiveness of base fluid heat transfer, resulting in enhanced thermal conductivity. But base fluids have an extremely low thermophysical phenomenon. They have distinct physical and chemical features. In the last decades, a lot of research has been performed on heat transfer convection, modeling, and their programs. Eid (2022) investigated the magnetic rotating hybridizing nanoliquid flow over a solar collector with the Cattaneo–Christov heat flux (CCHF) theory and centripetal and Coriolis forces. Shahzad et al. (2022) examined Burger’s nanoliquid circulation with motile organisms and CCHF. Ghalambaz et al. (2015) scrutinized the impact of nanoparticle diameter and natural convection on a vertical cone in the presence of the porous medium. Veera Krishna and Chamkha (2019) studied the effects of hall and ion slip on the nanofluid rotating boundary layer flow across an infinite vertical plate encased in a porous medium. Reddy et al. (2017) considered the magnetohydrodynamic boundary layer flow of a rotating disk in the presence of the porous medium with nanofluid flow.

The NF exhibit a low heat transfer. So, in the context of NF, more than one nanosized particle is embedded with the base liquid, resulting in a hybrid nanofluid (HNF). When compared to NFs, HNFs have higher thermal conductivity. Over the past few decades, HNFs are constantly being studied experimentally and numerically. Khan et al. (2020) discussed the heat radiation upshot on the mixed convective movement of an HNF. Nisar et al. (2020) scrutinized the convective stream of an HNF with the suspension of magnetized ferroparticles between multiple disks. Madhukesh et al. (2021a) numerically examined the HNF flow across a curved sheet with stretching. Manohar et al. (2021) investigated the role of HNF *via* a semi-spherical permeable fin. Sreedevi et al. (2020) examined the analysis of unstable hybrid nanofluid flow across a stretched sheet with thermal radiation in terms of mass and heat transmission. Some of the useful works conducted on HNF are listed in Jamshed et al. (2022) and Sajid et al. (2022).

Porous media are solid materials made up of porous structures, which are often filled with fluid in biological applications. In fluid mechanics, the porous medium can be regarded as a solid structure with fluid-flowing channels. Engineering applications for porous medium heat transfer difficulties include separation processes in chemical industries, geothermal energy extraction, thermal energy storage, crude oil extraction, transpiration cooling, groundwater contamination, and fiber insulation. Umeshaiah et al. (2022) employed a porous medium to inspect the flow of dusty NF over a stretched cylinder with melting. Madhukesh et al. (2021b) analyzed the flow of Casson NF through a porous medium. Ramesh et al. (2022) used ternary NF to study the HSS and porosity effects in a stretched divergent/convergent channel. Chamkha and Ben-Nakhi (2008) examined the presence of Soret and Dufour effects and MHD mixed convection–radiation interaction along a permeable surface submerged in a porous medium. MHD heat and mass transfer oscillatory flow of a micropolar fluid across a vertical permeable plate in a porous medium through an analytical investigation was carried out by Modather et al. (2009).

Chemical reactions, both heterogeneous and homogeneous (H-H) are, especially important since many chemically reacting systems incorporate both H-H reactions, such as fog dispersion, cooling towers, biological systems, cooling towers, catalysis, and hydrometallurgical processes. The connection amid homogeneous processes in the bulk of the liquid and heterogeneous reactions on specific catalytic surfaces is fairly intricate. So, there is a three-way interaction among surface/liquid temperatures, and fluid and reactant species concentrations. Siddiqui et al. (2022) swotted the effect of H-H processes on the 3D flow of water-based NFs as well as the estimation of entropy generation. Mahato et al. (2022) explored the role of radiation and inclined magnetic field upshot in the stream of NFs with H-H reactions. Khashi’ie et al. (2021) educed the stream of a radiative HNF across a permeable shrinking/stretching sheet with a H-H reaction. Waseem et al. (2021) investigated the hydromagnetic stream of couple stress NHF past a heated plate using homogeneous–heterogeneous reactions. Rooman et al. (2021) used the Hall effect across a rotating disk to optimize the entropy of Jeffrey NF flow with H-H reactions.

Thermal conductivity, material thickness, specific heat capacity, flow rate, and other components all have an impact on heat transmission in heat exchange. Heat source/sink (HSS): a heat source is anything that generates or emits heat. Using a passive heat exchanger called a heat sink, heat from any liquids is transferred into a flowing cooling liquid. When an HSS is utilized, the heat distribution all over the entire field changes. It disperses the system’s surplus energy. Magyari and Chamkha (2010) scrutinized the combined impact of heat production or absorption and first-order chemical reaction on micropolar fluid flows across a uniformly stretched permeable surface. Madhukesh et al. (2022) investigated the dynamics of water-based NF with swimming microbes across a Riga sheet that was constrained to a heat source/sink. Ramesh et al. (2020) conferred the impact of aluminum alloy and magnetite graphene oxide heat transfer investigation through a permeability cylinder with a heat source/sink. Ahmed et al. (2019) discussed Maxwell NF flow across a porous radially shrinking/stretching rotating disk. Hayat et al. (2016) conducted extensive research on the HSS features in NF flow along with nonlinear thermal radiation.

Due to its wide range of uses in science and industry, scientists are paying special attention to fluid stream across many geometries, including a vertical plate, cone, and wedge, among many other geometries. Many scientists and scholars have analyzed this issue from diverse perspectives. Furthermore, there are several applications utilizing these geometries such as NF flow heat transport *via* plates and wedges. Recently, Rekha et al. (2022) carried out research on the flow of HNF *via* a wedge, cone, and plate. Chamkha (1996) scrutinized non-Darcy hydromagnetic free convection in porous media in the presence of a cone and a wedge. Hussain et al. (2022) swotted the stream and thermal properties of a moving stretched porous wedge in MHD Casson NF. Veera Krishna et al. (2020) pondered the exponentially accelerating plate and educed the unstable MHD rotating stream over a saturated porous material. Al-Harbi (2005) provided numerical research on natural convective heat transfer from a wedge and a cone with changing thermal radiation and viscosity.

According to the aforementioned literature, no research has been conducted on the HNF flow across three distinct geometries when a porous medium, H-H reactions, and HSS are present. The current work is to investigate the influence of the porous medium, H-H reactions, and HSS in the presence of HNF flow on three different geometries. The current investigation is carried out to find the answers to the following questions:

1) What is the role of the porous parameter in the flow profile?
2) What is the influence of solid volume fraction on the rate of thermal distribution?
3) What happens to the concentration profile when the values of homogeneous and heterogeneous parameter values are increased?

The current investigation can be useful in applications like thermal transportation *via* nanofluid over different geometries like a plate and wedge, and this impact can be used in fog dispersion, cooling towers, biological systems, catalysis, and hydrometallurgical processes. The current investigation can be extended to investigate thermal transportation by considering ternary nanoparticles/various non-Newtonian fluids in the presence of a non-uniform heat source/sink, zero mass flux conditions with the combination of concentration, and bioconvection.

## Formulation

**FIGURE 1:**
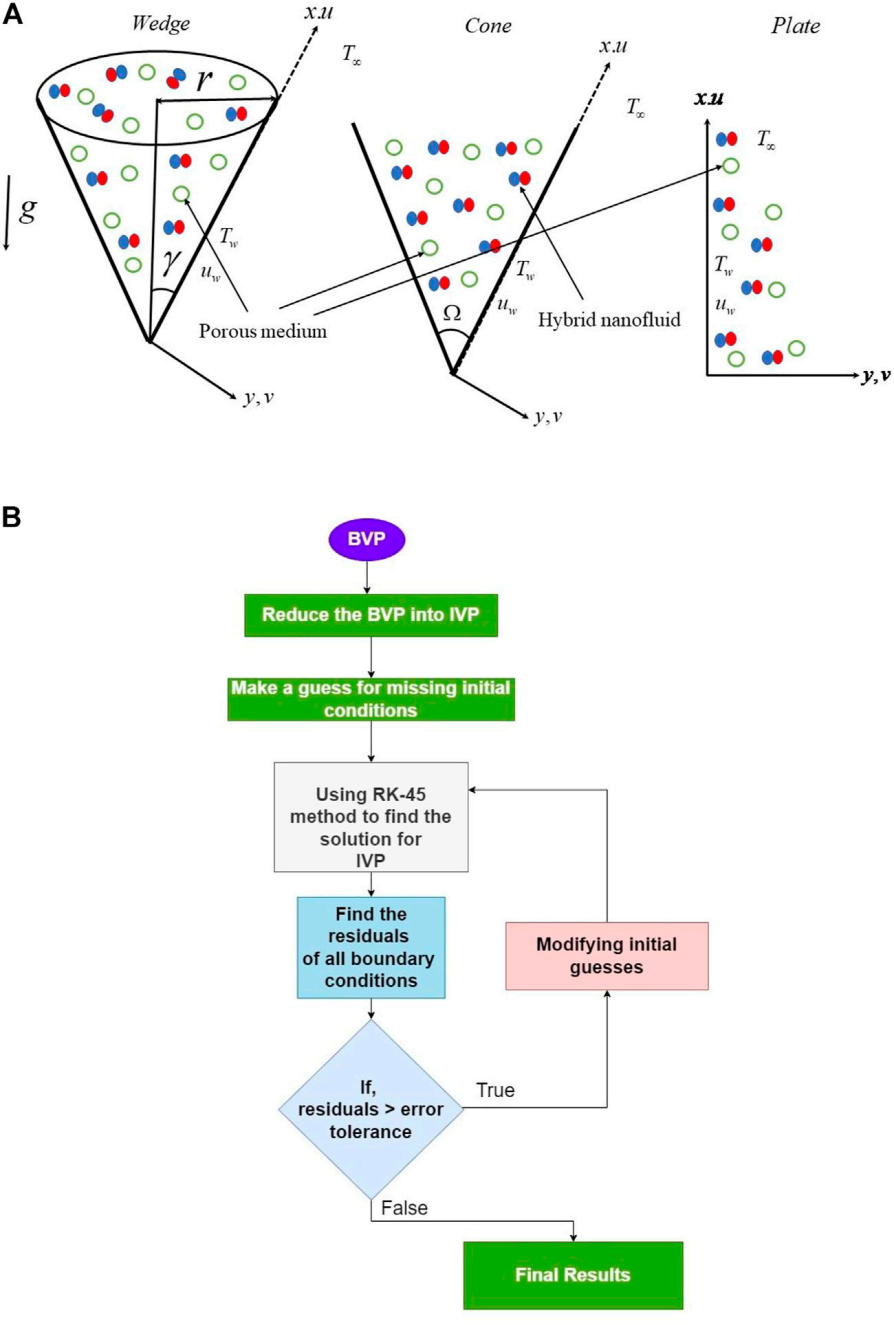
**(A)** Flow geometry. **(B)** Flow chart of the numerical scheme.

An incompressible HNF flow across wedge, cone, and plate geometries with a porous medium, HSS, and H–H reactions is considered. The coordinate *x*-axis is taken along the surface of the body, and y is normal to its surface. Figure 1A illustrates the model’s physical manifestation. Let us assume $\left(\gamma_{1},\Omega ,r\right)$ is the half-angle of the cone/wedge, full angle of the wedge, and radius of the cone, respectively. Let us assume that $T_{\infty }$ is the far-field temperature, $T_{w}$ is the temperature near the surface, $C_{w}$ is the concentration near the surface, and $C_{\infty }$ is the far-field concentration.

The isothermal cubic autocatalytic model for H-H reactions involving two chemical species $A_{1}$ and $B_{1}$ , proposed by Merkin (1996) and Chaudhary and Merkin (1995a); Chaudhary and Merkin (1995b), is given by

$$A_{1}+2B_{1}\to 3B_{1}\;rate=k_{c}ab^{2}$$

$$A_{1}\to B_{1}\;rate=k_{s}a$$

Here, the concentration of the chemical species $A_{1}\;,\;B_{1}$ is given by a ,b , respectively. $k_{i},\left(i=c,s\right)$ denotes the rate quantities. Both types of reactions are termed isothermal. Therefore, in Chamkha (1996), Chamkha (1997), and Chamkha and Ben-Nakhi (2008), the non-Darcy effect associated with the porous media inertia effect was considered to be infinitesimal and hence ignored. Furthermore, based on the aforementioned conditions, the equations for continuity, momentum, temperature, and concentration are as follows (Vajravelu and Nayfeh, 1992; Mahdy, 2019):

$$\frac{\partial \left(r^{n_{3}}u\right)}{\partial x}+\frac{\partial \left(r^{n_{3}}v\right)}{\partial y}=0,$$ (1)

$$u\frac{\partial u}{\partial x}+v\frac{\partial u}{\partial y}=\nu_{hnf}\frac{\partial^{2}u}{\partial y^{2}}+\frac{g\left(T-T_{\infty }\right){\left(\beta \rho \right)}_{hnf}\cos\gamma_{1}}{\rho_{hnf}}-\nu_{hnf}\frac{u}{K^{*}},$$ (2)

$$v\frac{\partial T}{\partial y}+u\frac{\partial T}{\partial x}=\frac{k_{hnf}}{{\left(\rho Cp\right)}_{hnf}}\frac{\partial^{2}T}{\partial y^{2}}+\frac{Q_{1}}{{\left(\rho Cp\right)}_{hnf}}\left(T-T_{\infty }\right),$$ (3)

$$v\frac{\partial a}{\partial y}+u\frac{\partial a}{\partial x}=D_{A}\frac{\partial^{2}a}{\partial y^{2}}-k_{c}ab^{2},$$ (4)

$$v\frac{\partial b}{\partial y}+u\frac{\partial b}{\partial x}=D_{B}\frac{\partial^{2}b}{\partial y^{2}}+k_{c}ab^{2},$$ (5)

with boundary conditions (Ali and Sandeep, 2017;Devi and Devi, 2016):

$$\begin{array}{l} u=u_{w}=\frac{\nu_{f}x}{l^{2}},\;\\ T=T_{w},\\ v=0,\\ D_{A}\frac{\partial a}{\partial y}=lk_{s}a,\\ D_{B}\frac{\partial b}{\partial y}=-lk_{s}a, \end{array}\}at\;y=0\;and\begin{array}{l} u\to 0,\\ T\to T_{\infty },\\ a\to a_{0,}\\ b\to 0\;, \end{array}\}as\;y\to \infty .$$ (6)

From the aforementioned expressions, $u\&v\left(ms^{-1}\right)$ denotes the velocity components along x and y (m) directions. $g\left(ms^{-2}\right)$ is acceleration due to gravity; $\beta \left(K^{-1}\right)$ is the thermal expansion factor; $K^{*}$ is permeability of the porous medium; $\nu_{f}\left(=\mu_{f}/\rho_{f}\right)$ is kinematic viscosity; $Q_{1}$ $\left(kgm^{-1}s^{-3}K^{-1}\right)$ is the rate of heat generation/absorption; a and b are the concentration of the chemical species $A_{1}\;,\;B_{1}$ ; $D_{A}$ and $D_{B}\left(m^{2}s^{-1}\right)$ are the diffusivity; $\rho_{f}\left(kgm^{-3}\right)$ is the density; $\mu \left(kgm^{-1}s^{-1}\right)$ is the dynamic viscosity; T , $T_{w}$ , and $T_{\infty }$ (K) denotes temperature, wall temperature, and ambient temperature, respectively; $n_{3}$ is the geometric factor; $Cp\left(m^{2}s^{-2}K^{-1}\right)$ is the specific heat; and the subscript hnf denotes hybrid nanofluid.

The proposed issue provides three different geometries based on the following hypotheses:

1) Case 1: wedge $—n_{3}=0$ and $\gamma_{1}\neq 0$ .
2) Case 2: cone $—n_{3}=1$ and $\gamma_{1}\neq 0$ .
3) Case 3: plate $—n_{3}=0$ and $\gamma_{1}=0$ .

Following similarity variables are introduced:

$$\begin{array}{c} u=\frac{\nu_{f}x}{l^{2}}f^{\prime },\\ v=\frac{-\left(n_{3}+1\right)\nu_{f}}{l}f,\\ \eta =\frac{y}{l},\\ \theta =\frac{T-T_{\infty }}{T_{w}-T_{\infty }},\\ \chi_{1}=\frac{a}{a_{0}},\\ \chi_{2}=\frac{b}{a_{0}}. \end{array}$$ (7)

The thermophysical properties of base fluid and nanoparticles are given in Table 1 (Devi and Devi, 2016). Furthermore, the correlations of the hybrid nanofluid are given as follows:

$$\rho_{hnf}=\left[\left(1-\phi_{1}\right)\rho_{f}+\phi_{1}\rho_{1}\right]\left(1-\phi_{2}\right)+\rho_{2}\phi_{2},$$ (8)

$$\mu_{hnf}=\frac{\mu_{f}}{{\left(1-\phi_{1}\right)}^{2.5}{\left(1-\phi_{2}\right)}^{2.5}},$$ (9)

$$k_{hnf}=\frac{2k_{nf}+k_{2}+\left(k_{2}-k_{nf}\right)2\phi_{2}}{2k_{nf}+k_{2}-\phi_{2}\left(k_{2}-k_{nf}\right)}k_{nf}\;and\;k_{nf}=\frac{k_{1}+2k_{f}-2\phi_{1}\left(k_{f}-k_{1}\right)}{k_{1}+2k_{f}+\phi_{1}\left(k_{f}-k_{1}\right)}k_{f},$$ (10)

$${\left(\rho C_{p}\right)}_{hnf}=\left[\left(1-\phi_{1}\right){\left(\rho C_{p}\right)}_{f}+\phi_{1}{\left(\rho C_{p}\right)}_{1}\right]\left(1-\phi_{2}\right)+{\left(\rho C_{p}\right)}_{2}\phi_{2},$$ (11)

$${\left(\rho \beta \right)}_{hnf}=\left[\left(1-\phi_{1}\right){\left(\rho \beta \right)}_{f}+\phi_{1}{\left(\rho \beta \right)}_{1}\right]\left(1-\phi_{2}\right)+{\left(\rho \beta \right)}_{2}\phi_{2}.$$ (12)

**TABLE 1:** Thermophysical properties of the base fluid and nanoparticle (Rana and Bhargava, 2011).

Property	k	$C_{p}$	ρ	$\beta_{T}\;\left(\times 10^{-5}\right)$
Al_2_O_3_	40	765	3970	0.85
Water	0.613	4179	997.1	21
CuO	76.5	531.8	6320	1.8

Here, the aforementioned Eqs 8–12 are used for the thermophysical features of the base fluid and the hybrid nanoparticles. The symbol ϕ corresponds to the solid nanoparticle volume fractions, and furthermore it is equal to the sum of two dissimilar nanoparticles $\phi_{1}$ (alumina) and $\phi_{2}$ (copper oxide). Meanwhile, the special case ϕ=0 corresponds to the normal base fluid, and the subscripts 1 and 2 denote the solid nanoparticles, hnf signifies the hybrid nanofluid, and f denotes the normal base fluid.

After utilizing the similarity transformations, the following reduced equations are expressed:

$$\frac{f^{\prime \prime \prime }}{\zeta_{1}\zeta_{2}}+f^{\prime \prime }f\left(n_{3}+1\right)-{\left(f^{\prime }\right)}^{2}+\frac{\zeta_{4}Gr\theta \cos\gamma_{1}}{\zeta_{2}}-\frac{\left(\lambda \right)f^{\prime }}{\zeta_{1}\zeta_{2}}=0,$$ (13)

$$\frac{k_{hnf}}{k_{f}}\frac{\theta^{\prime \prime }}{\mathrm{Pr}\zeta_{3}}+\left(n_{3}+1\right)f\theta^{\prime }+\frac{Hs\theta }{\zeta_{3}}=0,$$ (14)

$$\frac{\chi_{1}^{\prime \prime }}{Sc}+\left(n_{3}+1\right)f\chi_{1}^{\prime }-Kc\chi_{1}\chi_{2}^{2}=0,$$ (15)

$$\frac{\delta \chi_{2}^{\prime \prime }}{Sc}+\left(n_{3}+1\right)f\chi_{2}^{\prime }+Kc\chi_{1}\chi_{2}^{2}=0.$$ (16)

We predict the diffusion coefficients of $A_{1}$ and $B_{1}$ to be equal in most situations. As a result, we must also assume that the diffusion factors $D_{A}$ and $D_{B}$ are identical, and this leads to δ=1 (Chaudhary and Merkin, 1995a; Chaudhary and Merkin, 1995b). In this case, we get, $\chi_{1}+\chi_{2}=1$ . Thus, Eqs 15, 16 reduce to the form as follows:

$$\frac{\chi_{1}^{\prime \prime }}{Sc}+\left(n_{3}+1\right)f\chi_{1}^{\prime }-Kc\chi_{1}{\left(1-\chi_{1}\right)}^{2}=0,$$ (17)

and the boundary conditions in the reduced form are:

$$\begin{array}{l} \theta \left(0\right)=f^{\prime }\left(0\right)=1,\;f\left(0\right)=0\;,\chi_{1}^{\prime }\left(0\right)=Ks\chi_{1}\;\left(0\right),\;at\;\eta =0,\\ f^{\prime }\left(\infty \right)=\theta \left(\infty \right)=0,\chi_{1}\;\left(\infty \right)=1,\;as\;\eta \to \infty \end{array}\}$$ (18)

in which:

$$\begin{array}{c} \zeta_{1}={\left(1-\phi_{1}\right)}^{2.5}{\left(1-\phi_{2}\right)}^{2.5},\\ \zeta_{2}=\left\{\left(1-\phi_{2}\right)\left[\left(1-\phi_{1}\right)+\phi_{1}\frac{\rho_{1}}{\rho_{f}}\right]+\phi_{2}\frac{\rho_{2}}{\rho_{f}}\right\},\\ \zeta_{3}=\left(1-\phi_{2}\right)\left[\left(1-\phi_{1}\right)+\phi_{1}\frac{{\left(\rho C_{p}\right)}_{1}}{{\left(\rho C_{p}\right)}_{f}}\right]+\phi_{2}\frac{{\left(\rho C_{p}\right)}_{2}}{{\left(\rho C_{p}\right)}_{f}},\\ \zeta_{4}=\left(1-\phi_{2}\right)\left[\left(1-\phi_{1}\right)+\phi_{1}\frac{{\left(\rho \beta_{T}\right)}_{1}}{{\left(\rho \beta_{T}\right)}_{f}}\right]+\phi_{2}\frac{{\left(\rho \beta_{T}\right)}_{2}}{{\left(\rho \beta_{T}\right)}_{f}}. \end{array}$$ (19)

The aforementioned equations required similarity equations comprising the distinct controlling influential constraints, which are listed as follows: $Gr=\frac{g\beta \left(T_{w}-T_{\infty }\right)l^{2}}{u_{w}\nu_{f}}$ is the Grashof number, $\lambda =\frac{l^{2}}{K^{*}}$ is the porous parameter, $\mathrm{Pr}=\frac{\left(\rho Cp\right)\nu_{f}}{k}$ is the Prandtl number, $Sc=\frac{\nu_{f}}{D_{A}}$ is the Schmidt number, $Hs=\frac{Q_{1}l^{2}}{{\left(\rho Cp\right)}_{f}\nu_{f}}$ is the heat source/sink parameter, $Kc=\frac{k_{c}a_{0}^{2}l^{2}}{\nu_{f}}$ is the homogeneous reaction strength, $Ks=\frac{k_{s}l^{2}}{D_{A}}$ is the heterogeneous reaction strength, $\delta =\frac{D_{B}}{D_{A}}$ is the ratio of diffusion coefficient species, and $n_{3}$ is the geometric factor.

The important engineering factors and its reduced forms are given by Vajravelu and Nayfeh (1992) as follows:

$$Cf^{*}=\frac{\tau_{w}}{u_{w}^{2}\rho_{f}},Nu=\frac{q_{w}{\left(T_{w}-T_{\infty }\right)}^{-1}}{k_{f}l^{-1}},and\;Sh=\frac{j_{w}{\left(C_{w}-C_{\infty }\right)}^{-1}}{D_{B}l^{-1}}.$$ (20)

$$\mathrm{Here},\;\tau_{w}=\mu_{hnf}{\frac{\partial u}{\partial y}|}_{y=0},q_{w}=-{k_{hnf}\frac{\partial T}{\partial y}|}_{y=0},and\;j_{w}=-{D_{B}\frac{\partial C}{\partial y}|}_{y=0}.$$ (21)

Utilizing similarity variables and Eq. 20 into (19), the resultant equations are given in Eq. 21:

$$Cf=\frac{l}{x}Cf^{*}=\frac{f^{\prime \prime }\left(0\right)}{\zeta_{1}},Nu=\frac{-\theta^{\prime }\left(0\right)k_{hnf}}{k_{f}},and\;Sh=-\chi^{\prime }\left(0\right).$$ (22)

## Numerical scheme and validation of the code

The shooting strategy was used to solve ODEs (13, 14, and 17) with associated constraints (18) using the RKF 45 order approach. The acquired ODEs, together with the boundary constraints, are turned into initial value problems in order to solve these equations (IVP). For this, we choose,

$$f=c_{1},f^{\prime }=c_{2},f^{\prime \prime }=c_{3},\theta =c_{4},\theta \prime =c_{5},\chi_{1}=c_{6},\chi_{1}\prime =c_{7,}$$ (23)

$$f^{\prime \prime \prime }=-\zeta_{1}\zeta_{2}\left(c_{3}c_{1}\left(n_{3}+1\right)-{\left(c_{2}\right)}^{2}+\frac{\zeta_{4}Grc_{4}\cos\gamma_{1}}{\zeta_{2}}-\frac{\left(\lambda \right)c_{2}}{\zeta_{1}\zeta_{2}}\right),$$ (24)

$$\theta^{\prime \prime }=-\frac{\mathrm{Pr}\zeta_{3}k_{f}}{k_{hnf}}\left(\left(n_{3}+1\right)c_{1}c_{5}+\frac{Hsc_{4}}{\zeta_{3}}\right),$$ (25)

$$\chi_{1}^{\prime \prime }=-Sc\left(\left(n_{3}+1\right)c_{1}c_{7}-Kcc_{6}{\left(1-c_{6}\right)}^{2}\right),$$ (26)

and with known and unknown initial conditions becoming

$$\begin{array}{l} c_{1}\left(0\right)=0\;,\\ c_{2}\left(0\right)=1,\\ c_{3}\left(0\right)=e_{1},\\ c_{4}\left(0\right)=1,\\ c_{5}\left(0\right)=e_{2}\;,\\ c_{6}\left(0\right)=e_{3},\;\\ \frac{c_{7}\left(0\right)}{c_{6}\;\left(0\right)}=Ks.\; \end{array}\}$$ (27)

The equations which are simplified to the first order are numerically solved with the help of the RKF-45 method by guessing the missing boundary values with the help of a shooting procedure by choosing the parameter values as Gr=λ=1,Hs=Kc=Ks=0.1,Sc=0.8 with a calculation step size 0.1, mesh size is about 100, and convergence criteria are chosen nearly 9, so that the solution converges asymptotically with an error tolerance of roughly about $10^{-6}$ . The present numerical code is validated with the available literature by limiting the values of the constraints. Figure 1B displays the flow chart of the numerical scheme.

The algorithm of the RKF-45 method is as follows:

$$\begin{array}{c} a_{1}=h_{1}f_{1}\left(t_{1k},y_{1k}\right),\\ a_{2}=h_{1}f_{1}\left(t_{1k}+\frac{1}{4}h_{1},y_{1k}+\frac{1}{4}a_{1}\right),\\ a_{3}=h_{1}f_{1}\left(t_{1k}+\frac{3}{8}h_{1},y_{1k}+\frac{3}{32}a_{1}+\frac{9}{32}a_{2}\right),\\ a_{4}=h_{1}f_{1}\left(t_{1k}+\frac{12}{13}h_{1},y_{1k}+\frac{1932}{2197}a_{1}-\frac{7200}{2197}a_{2}+\frac{7296}{2197}a_{3}\right),\\ a_{5}=h_{1}f_{1}\left(t_{1k}+h_{1},y_{1k}+\frac{439}{216}a_{1}-8a_{2}+\frac{3680}{513}a_{3}-\frac{845}{4104}a_{4}\right),\\ a_{6}=h_{1}f_{1}\left(t_{1k}+\frac{1}{2}h_{1},y_{1k}-\frac{8}{27}a_{1}+2a_{2}-\frac{3544}{2565}a_{3}+\frac{1859}{4104}a_{4}-\frac{11}{40}a_{5}\right). \end{array}$$ (28)

Then, a Runge–Kutta technique of order 4 is used to approximate the IVP solution.

$$y_{1k+1}=y_{1k}+\frac{25}{216}a_{1}+\frac{1408}{2565}a_{3}+\frac{2197}{4101}a_{4}-\frac{1}{5}a_{5}.$$ (29)

A Runge–Kutta technique of order 5 is used to find a better value for the solution:

$$z_{1k+1}=y_{1k}+\frac{16}{135}a_{1}+\frac{6656}{12825}a_{3}+\frac{28561}{56430}a_{4}-\frac{9}{50}a_{5}+\frac{2}{55}a_{6}.$$ (30)

## Results and discussion

The properties of different dimensionless constraints on their corresponding profiles are described in detail in this section. The collection of governing equations is reduced into ODEs by employing apt similarity variables and thermophysical properties of nanoparticles mentioned in Table 1. The modified equations are numerically solved *via* the shooting approach and the RKF 45 process. The numerical findings are compared to the current works, and the best match is found (Table 2). Tables 3–5 demonstrate the main engineering factors on three different shapes in the manifestation of different constraints.

**TABLE 2:** Validation of the code for $-\theta^{\prime }\left(0\right)$ with respect to some reduced cases.

Pr	Salleh et al. (2010)	Present results
0.72	0.46317	0.46347
1	0.58198	0.58200
3	1.16522	1.16527
5	1.56806	1.56809
7	1.89548	1.89553
10	2.30821	2.30837
100	7.76249	7.76259

**TABLE 3:** Change in $f^{\prime \prime }\left(0\right)$ and $\theta^{\prime }\left(0\right)$ for various parameters in the cone when Sc=0.8,Kc=Ks=0.1 .

Parameter			$-f^{\prime \prime }\left(0\right)$			$-\theta^{\prime }\left(0\right)$		
Gr	λ	Hs	$\begin{array}{l} \phi_{1}=0.01\\ \phi_{2}=0.01 \end{array}$	$\begin{array}{l} \phi_{1}=0.01\\ \phi_{2}=0 \end{array}$	$\phi_{1}=\phi_{2}=0$	$\begin{array}{l} \phi_{1}=0.01\\ \phi_{2}=0.01 \end{array}$	$\begin{array}{l} \phi_{1}=0.01\\ \phi_{2}=0 \end{array}$	$\phi_{1}=\phi_{2}=0$
1	1	0.1	1.400283	1.410429	1.419443	1.862376	1.897508	1.900342
5	—	—	0.969792	0.975524	0.975108	1.932668	1.968334	0.975108
10	—	—	0.465046	0.465352	0.454325	2.004684	2.041042	0.454325
	1	—	1.400283	1.410429	1.419443	1.862376	1.897508	1.900342
	1.5	—	1.549492	1.562260	1.573953	1.827821	1.862350	1.864588
	2	—	1.687038	1.702108	1.716161	1.795784	1.829781	1.831492
	—	−0.5	1.432974	1.442571	1.451254	2.421712	2.463232	2.465713
	—	0	1.423294	1.432838	1.441331	1.959068	1.995329	1.998266
	—	0.5	1.408862	1.418364	1.426588	1.375103	1.405927	1.409734

**FIGURE 2:**
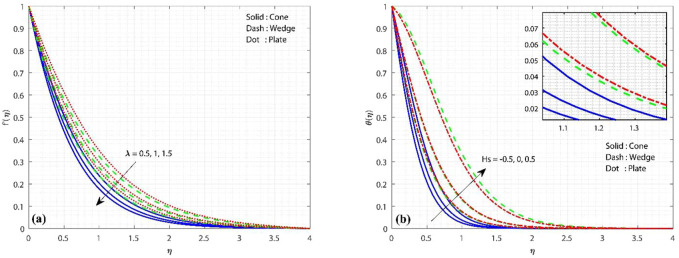
**(A)** Influence of porous constraint on the velocity profile, keeping Gr=1,Hs=Kc=Ks=0.1,Sc=0.8 constant, and **(B)** influence of the heat source/sink constraint on the temperature profile, keeping Gr=λ=1,Kc=Ks=0.1,Sc=0.8 constant.

It is well understood that the velocity at which fluid passes onto a certain surface is fully influenced by the distribution of velocity; hence, velocity dispersion plays a significant role in researching variations in the fluid flow rate. Figure 2A displays the nature of the porosity constraint over the velocity profile. The figure shows that the fluid motion declines as the porosity constraint enhances. This is due to the fact that the existence of a porous material will improve the size of the pores which restricts the fluid flow, causing it to decelerate and hence diminish velocity. Here, fluid velocity is high in the case of the plate and low in the case of the cone.

Figure 2B displays the variation in the thermal profile in the presence of Hs . The value of Hs=0 , Hs>0 , and Hs<0 denotes the absence of HSS, heat source, and heat sink, respectively. As the values of Hs improves, it gradually enlarges the thermal distribution in the system. This is owing to the fact that the outside surface of the geometry raises the temperature of the heat source. In the case of a heat sink, it acts as a heat exchanger, transferring the heat generated by the geometry’s surface into the fluid. From the diagram, it is clearly observed that thermal distribution is more in the case of the plate, and the least thermal distribution is observed in the cone.

**FIGURE 3:**
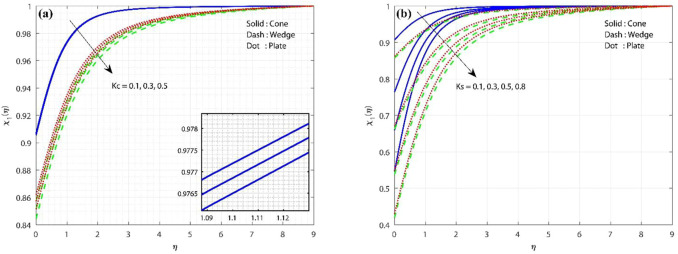
**(A)** Influence of homogeneous reaction strength on the concentration profile, keeping Gr=λ=1,Hs=Ks=0.1,Sc=0.8 constant, and **(B)** influence of heterogeneous reaction strength on the concentration profile, keeping Gr=λ=1,Hs=Kc=0.1,Sc=0.8 constant.

Figures 3A,B show the variations in H–H reaction strengths over the concentration profile. The increase in the value of Kc will diminish the mass transport, as shown in Figure 3A. The nanoscale particles and base liquid are in the same stage during a homogeneous reaction. In case of a heterogeneous reaction (reactions that occur on the surface of a distinct phase catalyst which improves the significant chemical reaction, which leads to reducing the chemical distribution in the flow system), a similar behavior is seen as in the homogeneous reaction. Figures 3A,B show that the concentration is lower in the wedge and higher in the cone in the presence of H-H reaction strengths.

**FIGURE 4:**
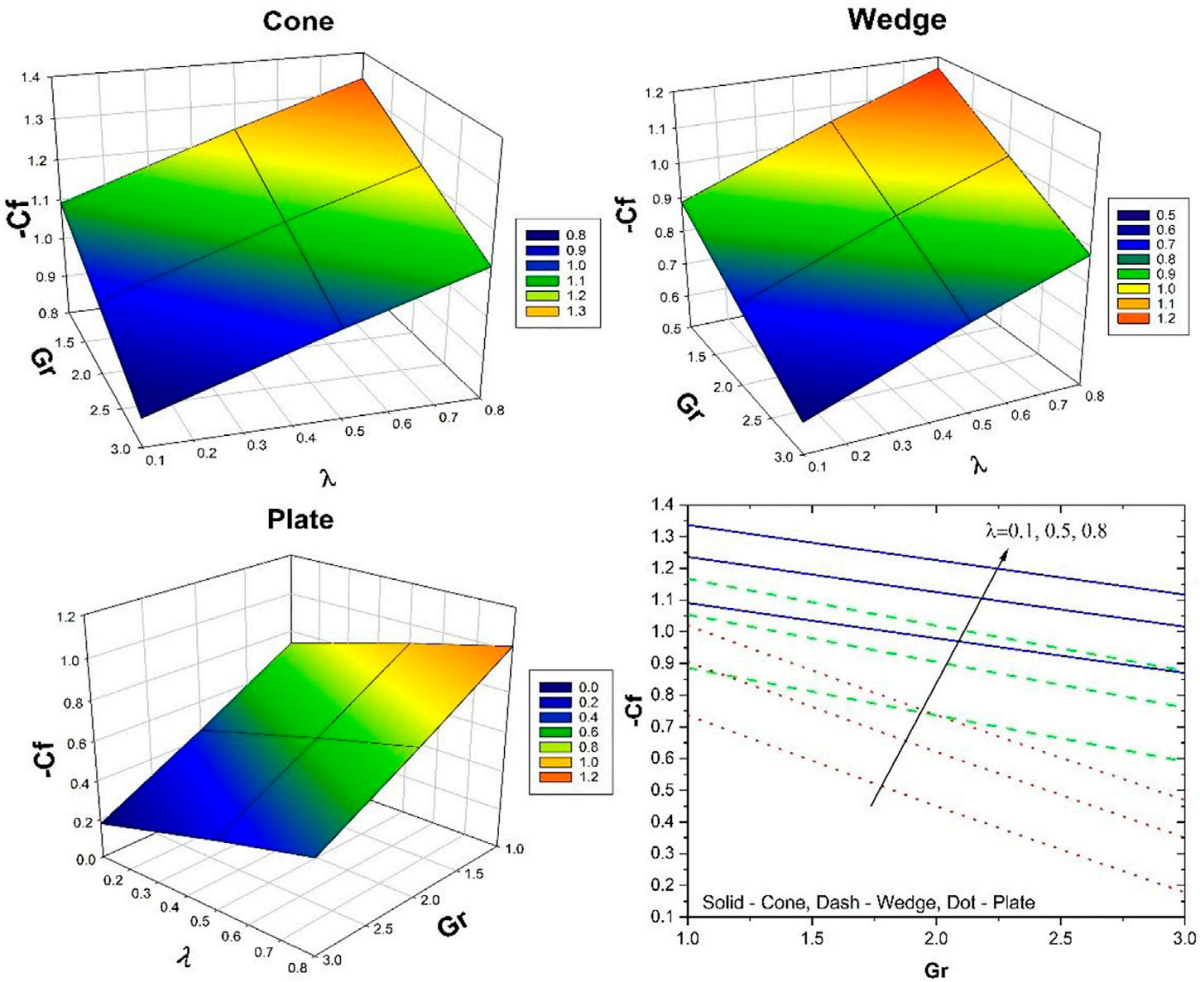
Behavior of −Cf over Gr for various values of λ.

The impact of −Cf on Gr for various values of λ is portrayed in Figure 4. Here, the surface drag force diminishes with increased Gr and λ . This is due to the increment of λ , the permeable medium improves which creates the drag force that opposes the movement of the liquid. As a result, −Cf decreases. −Cf is less in a cone and more in a plate.

**FIGURE 5:**
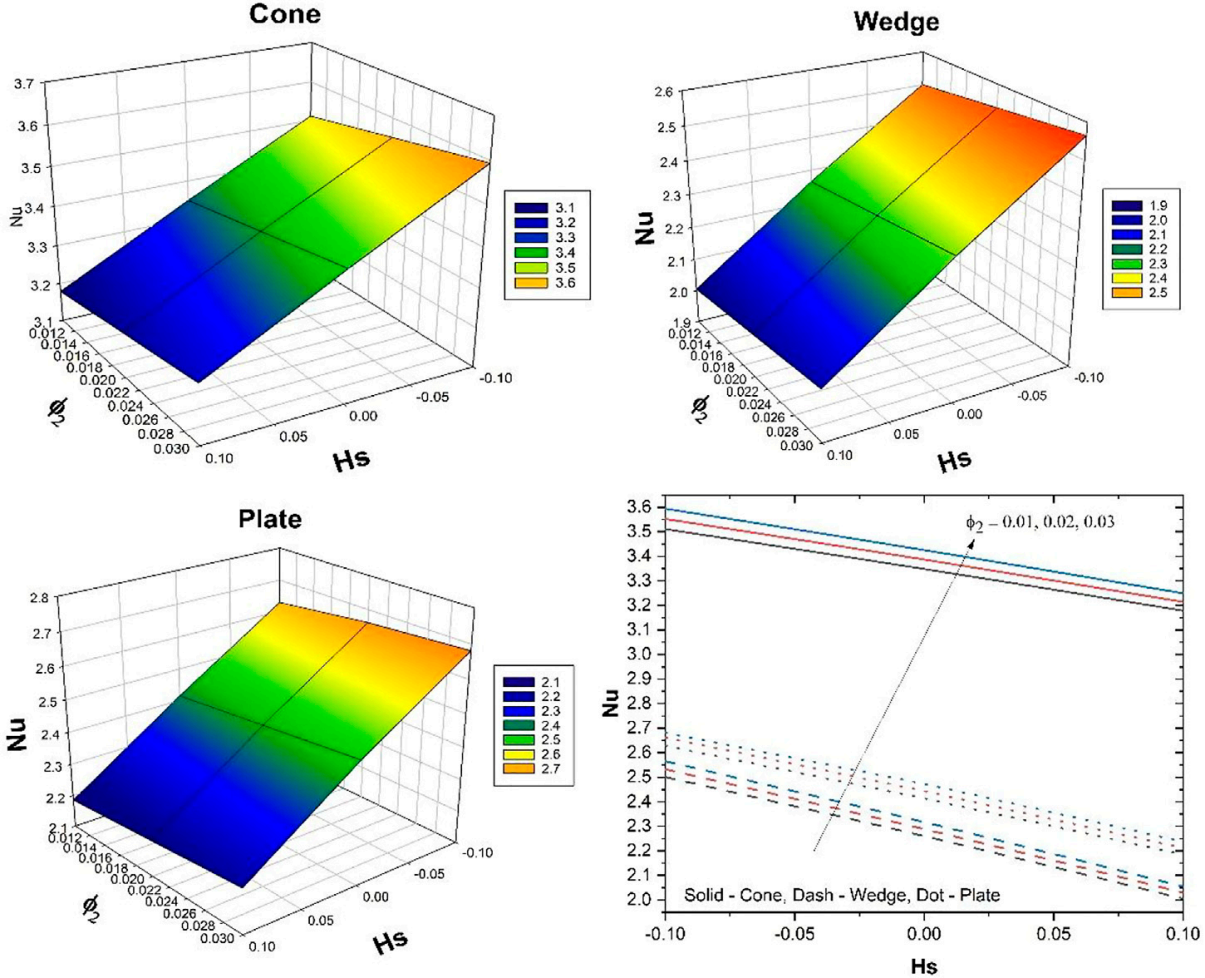
Behavior of Nu over Hs for various values of $\phi_{2}.$

The impact of Nu on Hs for various values of $\phi_{2}$ is illustrated in Figure 5. The rate of thermal distribution enhances with increased Hs and $\phi_{2}$ . The improvement in $\phi_{2}$ will thicken the thermal boundary layer, and an increase in Hs will improve the thermal distribution in the system. The rate of heat transport is more in the case of a cone and the least thermal distribution is seen in the case of a wedge.

Table 3 displays the variation in $f^{\prime \prime }\left(0\right)\&\theta^{\prime }\left(0\right)$ for Gr , λ , and Hs in cone geometry when Sc=0.8,Kc=Ks=0.1 for hybrid fluid, nanofluid, and viscous fluid. Here, the coefficients of skin friction show improvement in the presence of Gr and Hs , and a reverse trend is observed in the case of λ . $-\theta^{\prime }\left(0\right)$ enhances with larger values of Hs , but an opposite trend is established in Gr . The HNF plays a more prominent role than the NF and viscous fluid. The same behavior is observed in wedge and plate geometries (Tables 4, 5).

**TABLE 4:** Change in $f^{\prime \prime }\left(0\right)$ and $\theta^{\prime }\left(0\right)$ for various parameters in the wedge when Sc=0.8,Kc=Ks=0.1 .

Parameter			$-f^{\prime \prime }\left(0\right)$			$-\theta^{\prime }\left(0\right)$		
Gr	λ	Hs	$\begin{array}{l} \phi_{1}=0.01\\ \phi_{2}=0.01 \end{array}$	$\begin{array}{l} \phi_{1}=0.01\\ \phi_{2}=0 \end{array}$	$\phi_{1}=\phi_{2}=0$	$\begin{array}{l} \phi_{1}=0.01\\ \phi_{2}=0.01 \end{array}$	$\begin{array}{l} \phi_{1}=0.01\\ \phi_{2}=0 \end{array}$	$\phi_{1}=\phi_{2}=0$
1	1	0.1	1.238079	1.247846	1.256085	1.180946	1.204269	1.205930
5	—	—	0.675218	0.678814	0.675341	1.283103	1.307223	1.310849
10	—	—	0.038028	0.034280	0.0181181	1.373024	1.398114	1.403104
	1	—	1.238079	1.247846	1.256085	1.180946	1.204269	1.205930
	1.5	—	1.401688	1.414186	1.425265	1.138794	1.161443	1.162439
	2	—	1.550276	1.565133	1.578677	1.099999	1.122053	1.122469
	—	−0.5	1.296383	1.305425	1.313461	1.962916	1.995014	1.996376
	—	0	1.276035	1.284765	1.292401	1.347111	1.370614	1.372428
	—	0.5	1.233634	1.241449	1.248331	0.359555	0.366689	0.370300

**TABLE 5:** Change in $f^{\prime \prime }\left(0\right)\&\theta^{\prime }\left(0\right)$ for various parameters in the plate when Sc=0.8,Kc=Ks=0.1 .

Parameter			$-f^{\prime \prime }\left(0\right)$			$-\theta^{\prime }\left(0\right)$		
Gr	λ	Hs	$\begin{array}{l} \phi_{1}=0.01\\ \phi_{2}=0.01 \end{array}$	$\begin{array}{l} \phi_{1}=0.01\\ \phi_{2}=0 \end{array}$	$\phi_{1}=\phi_{2}=0$	$\begin{array}{l} \phi_{1}=0.01\\ \phi_{2}=0.01 \end{array}$	$\begin{array}{l} \phi_{1}=0.01\\ \phi_{2}=0 \end{array}$	$\phi_{1}=\phi_{2}=0$
1	1	0.1	1.090889	1.099093	1.104191	1.210755	1.234264	1.236564
5	—	—	0.038028	0.034281	0.018118	1.373024	1.398114	1.403105
10	—	—	−1.117223	−1.134630	−1.173186	1.501200	1.527898	1.534531
	1	—	1.090889	1.099093	1.104191	1.210755	1.234264	1.236564
	1.5	—	1.256682	1.267646	1.275685	1.170569	1.193411	1.195102
	2	—	1.407562	1.420912	1.431515	1.133601	1.155855	1.431515
	—	−0.5	1.175516	1.183083	1.188386	1.972300	2.004569	2.006161
	—	0	1.136729	1.143730	1.148311	1.367148	1.391100	1.393374
	—	0.5	1.060646	1.066209	1.069597	0.432229	0.441969	0.446885

## Conclusion

The current work looks into the upshot of the porous medium, H-H reactions, and HSS in the presence of HNF flow over three different geometries. Using similarity variables, the set of governing equations is converted into ODEs. Furthermore, these equations are numerically tackled with RKF-45 and shooting scheme. The important dimensionless constraints on their respective profiles are elucidated with the help of graphs. The important engineering coefficients are explained using tables. The major findings of the study are as follows:

1) The fluid velocity declines with improvement in the porous parameter.
2) The heat transport is more in the case of a heat source than a heat sink.
3) The H-H reaction strengths decline the concentration.
4) The fluid velocity and thermal distribution are high in plate geometry and less in cone geometry in the presence of λ and Hs , respectively.
5) The rate of thermal distribution increases with the increase in the solid volume fraction and heat source sink factor.
6) The surface drag force minimizes with the escalating values in the porous parameter.

## Data Availability

The original contributions presented in the study are included in the article/Supplementary Material; further inquiries can be directed to the corresponding author.

## Nomenclature

a and b: the concentration of the chemical species $A_{1}\;and\;B_{1}$
$A_{1}$ **and** $B_{1}$: chemical species w
$C_{w}$: concentration near the surface
$C_{\infty }$: far-field concentration
Cp: specific heat
Cf: skin friction $D_{A}$ and $D_{B}$ Diffusivity
f: normal base fluid
g: acceleration due to gravity
Gr: Grashof number
Hs: heat source/sink parameter
$K^{*}$: permeability of the porous medium
Kc: homogeneous reaction strength
Ks: heterogeneous reaction strength
$n_{3}$: geometric factor
Nu: Nusselt number
$Q_{1}$: Rate of heat generation/absorption
r: radius of the cone
Pr: Prandtl number
Sc: Schmidt number
Sh: Sherwood number
T: temperature
$T_{w}$: temperature near the surface
$T_{\infty }$: far-field temperature
u and v: velocity components
x and y: Cartesian coordinates
**Greek symbols:**
$\gamma_{1}$: half-angle of the cone/wedge
Ω: full-angle of the wedge
β: thermal expansion factor
$\nu_{f}$: kinematic viscosity
$\rho_{f}$: density
μ: dynamic viscosity
η: similarity variable
θ: dimensionless temperature profile
$\chi_{1}$ **and** $\chi_{2}$: dimensionless concentration profile
λ: porous parameter
δ: ratio of diffusion coefficient species
ϕ: solid nanoparticle
**Subscripts:**
hnf: hybrid nanofluid
$\phi_{1}$: alumina nanoparticle
$\phi_{2}$: copper oxide nanoparticle
